# Influence of clinical mastitis and its treatment outcome on reproductive performance in crossbred cows: A retrospective study

**DOI:** 10.14202/vetworld.2017.485-492

**Published:** 2017-05-07

**Authors:** Narender Kumar, A. Manimaran, M. Sivaram, A. Kumaresan, S. Jeyakumar, L. Sreela, P. Mooventhan, D. Rajendran

**Affiliations:** 1 Livestock Research Centre, Southern Regional Station, ICAR-National Dairy Research Institute (NDRI), Adugodi, Bengaluru - 560 030, Karnataka, India; 2 Dairy Economics and Statistics Section, Southern Regional Station, ICAR-National Dairy Research Institute (NDRI), Adugodi, Bengaluru - 560 030, Karnataka, India; 3 Theriogenology Laboratory, Animal Reproduction, Gynaecology & Obstetrics, Southern Regional Station, ICAR-National Dairy Research Institute (NDRI), Adugodi, Bengaluru - 560 030, Karnataka, India; 4 Livestock Production and Management Section, ICAR-National Dairy Research Institute (NDRI), Karnal - 132 001, Haryana, India; 5 ICAR-National Institute of Biotic Stress Management, Baronda, Raipur - 493 225, Chhattisgarh, India; 6 Division of Animal Nutrition, ICAR-National Institute of Animal Nutrition and Physiology, Adugodi, Bengaluru - 560 030, Karnataka, India

**Keywords:** clinical mastitis, crossbred cows, reproductive parameters, retrospective study

## Abstract

**Aim:**

Evaluation of the effect of clinical mastitis (CM) and its treatment outcome on the reproductive performance in crossbred cows retrospectively.

**Materials and Methods:**

Datasets of 835 lactating cows affected with CM during a period of 12 years (2001-2012) were considered for this study. Mastitis treatment related data and reproductive parameters such as days to first detected heat (DTFDH), days to first insemination (DTFI), days open (DO), and number of services per conception (SC) were collected from mastitis treatment and artificial insemination registers, respectively. Data were analyzed by ANOVA using SPSS 20 software. The means were compared with the Duncan’s multiple comparison post-hoc test.

**Results:**

CM affected cows had significantly (p<0.05) higher DTFDH, DTFI, DO and SC compared to clinically healthy cows. Cows diagnosed with a single episode of CM had significantly (p<0.05) delayed DTFDH while, DO and SC were significantly higher (p<0.05) in cows diagnosed by multiple episodes of CM. SC was significantly (p<0.05) higher in cows diagnosed with both relapse and recurrence. Severe CM affected cows had significantly (p<0.05) altered reproductive parameters. The reproductive parameters were altered to high extent when CM occurred during the breeding period.

**Conclusion:**

CM-affected cows had higher DTFDH, DTFI, DO and SC compared to clinically healthy cows. The negative effects of CM on reproduction parameters were higher when CM occurred during the breeding period.

## Introduction

Clinical mastitis (CM) is the most important disease in dairy cows leading to huge economic losses to dairy farmers through loss of milk yield and quality, and culling of CM affected animals. Earlier, the effect of mastitis was considered to be restricted to udder only except in severe cases where systemic illness was observed. However, off late, increasing evidence indicate that mastitis is also a critical factor affecting the reproductive success of the herd. Although a direct causal effect of mastitis on reproductive inefficiency has not been established, it is evident that a correlation between mammary gland health and fertility of lactating dairy cows exists [1]. One of the preliminary studies to demonstrate a correlation between the occurrence of mastitis and altered reproductive pattern of dairy cows was performed by Moore *et al*. [2] who reported an altered estrus interval in cows affected with mastitis. Later on, several researchers reported adverse effects of mastitis on reproduction based on retrospective data [3-6]. Higher incidence of embryonic loss or abortion in CM affected cows has also been reported [7,8].

In an extensive study, it was reported that the effects of CM on reproductive performance of dairy cows depend on the timing of occurrence of CM. Cows diagnosed with CM after 62 days postpartum (i.e., after voluntary waiting period) but before first artificial insemination (AI), had worse reproductive performance than cows that had CM during the early postpartum period [9]. Hertl *et al*. [10] and Lavon *et al*. [11] reported lower probability of conception in dairy cows diagnosed with CM at any time between 14 days before and 35 days after AI and this effect was less pronounced if the CM event occurred earlier than that while Hudson *et al*. [12] reported a prolonged risk period (28 days before and 70 days after AI) for the negative effect of CM on conception. These findings indicate that occurrence of CM during breeding period had negative impact on reproductive performance.

All these above cited studies were conducted on high milk producing, pure breed (*Bos taurus*) cows reared under temperate climate. On the contrary, effects of CM on reproduction performance of moderate producing crossbreds have not been studied in detail [9]. Since several developing countries are practicing crossbreeding to improve the milk production of native breeds, studying the effects of CM on reproduction assumes significance. Further, the effect of multiple episodes of CM occurrence on reproduction performance is not known well enough since most of the earlier studies reported only the effect of single episode of CM.

The present study was undertaken to analyze the effects of CM on reproduction performance of crossbred cows considering the number of episodes of CM, its severity, seasonality of occurrence, time of occurrence in relation to AI and parity of the cows. The reproduction parameters studied included days to first detected heat (DTFDH), days to first insemination (DTFI), days open (DO) and number of services per conception (SC).

## Materials and Methods

### Ethical approval

The present retrospective study was duly approved by the Institutional Animal Ethics Committee, ICAR - National Dairy Research Institute, Karnal, Haryana, India.

### Study site and data collection

This study was conducted on crossbred (Holstein Friesian × Tharparkar) cows maintained at Livestock Research Centre, National Dairy Research Institute, Karnal, India. The climate of the farm is subtropical in nature. The climate data records (1994-2004) of study site revealed that temperature humidity index (THI) was 58.77-68.62 during winter, 75.72-81.22 during summer, 77.19-77.67 during rainy, and 62.98-72.92 during autumn [13]. The air temperature varied from near freezing (4°C) in winter months to about 45°C in summer months. The average maximum (T_max_) and minimum (T_min_) temperature were 42.4°C and 3.3°C respectively, while the average maximum and minimum relative humidity were 92% and 67.8%, respectively. The average maximum and minimum THI were 86.5 and 63.75, respectively [14]. The average annual rainfall was approximately 760-960 mm. Datasets of 835 lactating cows affected with CM during a period of 12 years (2001-2012) were considered for this study. Clinically, healthy cows (n=187) and not diagnosed or treated against any clinical conditions during the same period was considered as control. The mastitis treatment related data and reproductive parameters of affected and control cows were collected from mastitis treatment and AI registers, respectively. Reproduction parameters such as DTFDH, DTFI, DO, and number of SC were calculated from AI register.

### Animals and management

The average number of lactating and dry animals in the herd during the observation period was 179 and 47, respectively. The average age at first calving was 34.5 months while average first lactation yield was 3142 kg. The all lactation yield for 305 days or less was 3583 kg with average milk yield of 12.3 kg/day. The average milk fat and solid not fat were 4.3% and 8.7% respectively, during 2001-2012. All animals were maintained in loose housing system under group management practice. The nutrient requirements of the animals were mostly met through *ad libitum* green fodder, dry fodder, silage and measured amount of concentrate at the rate of 1.5-2.0 kg per animal for body maintenance. Milking cows (yielding above 5.0 kg) were given additional concentrate at the rate of 1.0 kg for every 2.5 kg milk production as per the NRC recommendations [15].

After voluntary waiting period of 45-60 days, all the animals were routinely observed three times a day by trained personal for detection of estrus [16]. Animals identified to be in estrus were confirmed for their proper stage to receive semen by a trained veterinarian. Animals detected in estrus in the early morning were inseminated in morning of the same day with frozen semen and those cows detected in estrus in the late evening and night were inseminated in the morning of the next day. Two inseminations were done at 12 h intervals as per the routine procedure in the farm. Pregnancy diagnosis was performed 45-60 days after AI through rectal palpation.

### CM: Management and its classification

Three times milking (morning 5.00 to 6.00 am, noon 12.00 to 1.00 pm and in evening 6.00 to 7.00 pm) using milking machine was the routine practice in the farm. Before milking, pre-stripping and visual observation for CM were carried out by well-trained milkers. All the diagnosed to be affected with CM were immediately treated by veterinarian as per standard farm practices. Separate register for CM cases was maintained in the farm, in which information of animal identification number, quarter affected, symptoms, treatment details and date of clinical cure were mentioned. Cows calved during the study period, but not treated for any postpartum complication such as retained placenta, postpartum uterine infections such as metritis or endometritis and other metabolic disorders such as milk fever were considered as clinically healthy and included in the study. CM cases were defined as any abnormality in milk (watery, pus, blood, flakes, etc.), and udder tissue (swelling, hardness, pain, etc.) with or without systemic involvement (fever, anorexia, in appetence, dehydration, etc.). The absence of above symptoms and appearance of normal milk after treatment were considered as clinical cure. Episodes of CM was classified as single, relapse and recurrence. Relapse was defined as retreatment of the same animal within 21 days following clinical cure. Recurrence was defined as detection of a new CM episode in the same cow at least 21 days after treatment of the previous episode of CM. The severity of CM was classified into mild (only abnormality in milk), moderate (abnormality in milk and udder tissue), and severe (abnormality in milk, udder with systemic illness). Season was classified into four, viz., winter (December to March), summer (April to June), rainy (July to September), and autumn (October and November). To understand the effects of timing of CM occurrence on reproductive performance, it was classified as CM before first postpartum AI, CM after first postpartum AI but before pregnancy. To understand the critical period before first postpartum AI, they were further grouped into four groups: CM in the first 6 days in milk (DIM), CM between 7 and 29 DIM, CM between 30 and 62 DIM and CM over 62 DIM. Parities of the enrolled animals were classified as first, second, third, four and above four.

### Statistical analysis

To understand the effect of CM, the cows were grouped based on different type of episodes and severity while, to understand the influence of various factors among CM cows, they were grouped based on timing of CM occurrence, DIM before first AI, seasonality of CM occurrence and parities. The generated data were subjected to one-way ANOVA using SPSS 20. The significance mean between different categories were compared by Duncan multiple range test, and the results are expressed as mean±standard error of mean.

## Results

### Effect of CM on reproductive parameters

Effect of CM on reproductive parameters in crossbred cows is presented in Table-1. Mastitis affected cows had significantly (p<0.05) higher DTFDH, DTFI, DO and SC compared to clinically healthy cows.

**Table-1:** Effect of CM on reproduction parameters in crossbred cows.

Parameters	N	Mean±SE			

		DTFDH	DTFI	DO	SC
Control	187	61.67±1.44^a^	67.75±1.28^a^	73.44±1.26^a^	1.19±0.03^a^
Mastitis	835	73.87±1.37^b^	99.95±1.92^b^	164.99±3.59^b^	2.16±0.47^b^

Values with different superscripts within column differ significantly at p<0.05. SE=Standard error, CM=Clinical mastitis, DTFDH=Days to first detected heat, DTFI=Days to first insemination, DO=Days open, SC=Services per conception

### Effect of repeated or multiple episodes of CM on reproductive parameters

The cows diagnosed with a single episode of CM had significantly (p<0.05) higher DTFDH than multiple episode mastitic cows or clinically healthy cows while DO and SC were significantly higher (p<0.05) in cows diagnosed with multiple episodes compared to those single episode or clinically healthy cows (Table-2). Among the cows diagnosed with multiple episodes of CM, there was no significant influence of relapse, recurrence or combination of both on DTFDH and DTFI. However, the SC were significantly (p<0.05) higher in cows diagnosed with both relapse and recurrence of CM (Table-3).

**Table-2:** Effect of number of CM episodes (single and multiple) on reproduction parameters in crossbred cows.

Parameters	N	Mean±SE			

		DTFDH	DTFI	DO	SC
Control	187	61.67±1.44^a^	67.75±1.28^a^	73.44±1.26^a^	1.19±0.03^a^
Single	426	76.85±1.88^b^	100.38±2.54^b^	157.22±4.43^b^	2.02±0.06^b^
Multiple	409	70.68±2.00^c^	99.49±2.90^b^	173.32±5.67^c^	2.30±0.07^c^

Values with different superscripts within column differ significantly at p<0.05. SE=Standard error, CM=Clinical mastitis, DTFDH=Days to first detected heat, DTFI=Days to first insemination, DO=Days open, SC=Services per conception

**Table-3:** Effect of different types of CM episodes on reproduction parameters in crossbred cows.

Parameters	N	Mean±SE			

		DTFDH	DTFI	DO	SC
Control	187	61.67±1.44^a^	67.75±1.28^a^	73.44±1.26^a^	1.19±0.03^a^
Single	426	76.85±1.88^b^	100.38±2.54^b^	157.22±4.43^bc^	2.02±0.06^b^
Relapse	117	68.81±2.84^ab^	95.88±4.38^b^	152.96±7.98^b^	2.16±0.12^b^
Recurrence	141	76.13±3.40^b^	102.43±4.64^b^	173.13±8.67^c^	2.18±0.11^b^
Both relapse and recurrence	151	71.91±2.95^b^	98.87±5.33^b^	173.33±8.22^c^	2.48±0.13^c^

Values with different superscripts within column differ significantly at p<0.05. SE=Standard error, CM=Clinical mastitis, DTFDH=Days to first detected heat, DTFI=Days to first insemination, DO=Days open, SC=Services per conception

### Effect of severity of CM on reproductive parameters

A majority of the cows were affected with mild to moderately severe CM. There were no significant differences in reproductive parameters such as DTFDH, DTFI, DO and SC between cows affected with mild and moderate CM. However, severe CM affected cows had significantly (p<0.05) higher DTFDH, DTFI, DO and SC compared to cows affected with mild and moderate CM (Table-4).

**Table-4:** Effect of severity of CM on reproduction parameters in crossbred cows.

Severity level	N	Mean±SE			

		DTFDH	DTFI	DO	SC
Mild	557	71.30±1.81^a^	96.73±2.43^a^	158.38±4.02^a^	1.19±0.03^a^
Moderate	222	71.24±2.75^b^	96.40±3.83^a^	162.99±6.99^a^	2.02±0.06^a^
Severe	56	133.11±6.56^c^	152.68±12.23^b^	279±23.42^b^	2.30±0.07^b^

Values with different superscripts within column differ significantly at p<0.05. SE=Standard error, CM=Clinical mastitis, DTFDH=Days to first detected heat, DTFI=Days to first insemination, DO=Days open, SC=Services per conception

### Effects of timing of CM occurrence on reproductive parameters

Effects of timing of CM occurrence on reproductive parameters are presented in Table-5. DTFDH, DTFI, DO, and SC were significantly (p<0.05) higher when cows diagnosed with CM before or after first AI compared to healthy cows. The magnitude of adverse effects of CM on DO and SC were significantly higher in cows diagnosed with CM after first AI but before confirmation of pregnancy than the cows diagnosed with CM before first AI or healthy cows. We also assessed the effect of CM occurrence during different time period before first AI (i.e., during voluntary waiting period) on reproductive performance (Table-6) and found that occurrence of CM beyond 62 DIM had more (p<0.05) effects on DO and SC while CM occurrence during 30-62 DIM had minimum effect. Otherwise, all the fertility parameters were observed to be in similar trend during the voluntary waiting period.

**Table-5:** Effect of time of occurrence of CM on reproduction parameters in crossbred cows.

Parameters	N	Mean±SE			

		DTFDH	DTFI	DO	SC
Control	187	61.67±1.44^a^	67.75±1.28^a^	73.44±1.26^a^	1.19±0.03^a^
CM before first AI	550	74.21±1.94^b^	104.17±2.39^b^	160.92±4.38^b^	2.01±0.05^b^
CM after first AI but before pregnancy confirmation	98	82.15±5.70^b^	95.59±5.67^b^	269.34±12.66^c^	3.63±0.16^c^

Values with different superscripts within column differ significantly at p<0.05. AI=Artificial insemination, CM=Clinical mastitis, DTFDH=Days to first detected heat, DTFI=Days to first insemination, DO=Days open, SC=Services per conception, SE=Standard error

**Table-6:** Effect of the timing of CM occurrence before first service on reproduction parameters in crossbred cows.

DIM while CM	N	Mean±SE			

		DTFDH	DTFI	DO	SC
<7 days	278	71.54±3.26^ab^	93.67±2.89^ab^	149.64±5.68^ab^	1.97±0.07^a^
7 and 29 days	118	72.93±2.76^ab^	98.80±5.17^ab^	168.12±11.01^bc^	2.20±0.12^ab^
30 and 62 days	95	67.22±3.11^b^	91.96±4.81^b^	134.27±7.79^a^	1.91±0.12^a^
>62 days	344	82.76±3.24^a^	109.78±3.83^a^	191.43±6.35^c^	2.41±0.08^b^

Values with different superscripts within column differ significantly at p<0.05. DIM=Days in milk, CM=Clinical mastitis, DTFDH=Days to first detected heat, DTFI=Days to first insemination, DO=Days open, SC=Services per conception, SE=Standard error

### Effect of season of CM occurrence and parity of cows on reproductive parameters

We did not observe any significant effects of CM occurrence during different seasons on any of the fertility parameters (Table-7). We observed that the cows diagnosed with CM had significantly (p<0.05) higher DTFDH, DTFI, DO and SC in both primiparous and multiparous cows (Table-8). The DTFDH was significantly higher in first and second parity cows and no changes were observed in remaining parities.

**Table-7:** Effect of CM occurrence during different season on reproduction parameters in crossbred cows.

Season	N	Mean±SE			

		DTFDH	DTFI	DO	SC
Summer	208	77.03±3.44	103.13±4.16	167.27±6.90^ab^	2.20±0.09
Winter	329	74.91±3.75	103.99±3.98	179.20±6.62^b^	2.23±0.07
Rainy	200	75.08±2.78	94.33±3.61	151.47±7.00^a^	2.04±0.09
Autumn	98	74.52±3.27	94.93±4.45	163.31±10.38^ab^	2.22±0.15

Values with different superscripts within column differ significantly at p<0.05. CM=Clinical mastitis, DTFDH=Days to first detected heat, DTFI=Days to first insemination, DO=Days open, SC=Services per conception, SE=Standard error

**Table-8:** Effect of CM occurrence during different parity on reproduction parameters with parity in crossbred cows.

Parameters	N	Mean±SE			

		DTFDH	DTFI	DO	SC
1^st^ parity					
Control	70	59.47±2.31^a^	65.33±1.98^a^	69.23±1.96^a^	1.16±0.04^a^
Mastitis	255	85.14±3.925^b^	110.56±4.53^b^	176.04±7.14^b^	2.10±0.08^b^
2^nd^ parity					
Control	33	57.73±3.74^a^	67.36±3.03^a^	76.64±3.06^a^	1.24±0.07^a^
Mastitis	168	74.72±2.94^b^	104.80±5.02^b^	112.87±8.70^b^	2.21±0.10^b^
3^rd^ parity					
Control	27	72.07±3.93^a^	74.85±3.93^a^	82.00±3.83^a^	1.30±0.10^a^
Mastitis	136	73.79±2.61^a^	99.74±4.02^b^	173.17±9.63^b^	2.32±0.13^b^
4^th^ parity					
Control	14	61.57±6.27^a^	66.79±5.93^a^	73.93±4.56^a^	1.21±0.11^a^
Mastitis	104	73.00±3.51^a^	94.60±4.64^b^	162.17±9.37^b^	2.30±0.13^b^
>4 parity					
Control	43	61.79±2.40^a^	67.86±3.56^a^	72.33±2.27^a^	1.14±0.05^a^
Mastitis	172	66.63±5.04^a^	87.17±2.93^b^	147.48±7.56^b^	2.07±0.09^b^

Values with different superscripts within column differ significantly at p<0.05. CM=Clinical mastitis, DTFDH=Days to first detected heat, DTFI=Days to first insemination, DO=Days open, SC=Services per conception, SE=Standard error

## Discussion

Reproductive performance of dairy cows that experience mastitis could be affected in many ways. This study reports the effects of CM on economically important reproductive parameters in moderate producing crossbred cows reared under subtropical agro-eco system.

### Effect of CM on reproductive parameters

We observed that CM affected cows had significantly higher DTFDH, DTFI, DO and SC compared to clinically healthy cows. Delayed DTFDH in CM affected animals has been reported earlier [17]. Delayed DTFDH in CM affected cows might be due to altered hypothalamic–pituitary hormonal axis [18] and consequent onset of cyclicity [2]. Huszenicza *et al*. [19] also reported delayed onset of ovarian activity and estrus in CM affected cows. Our findings on DTFI and DO in CM affected cows are in agreement with those reported earlier [7,9,20]. In contrast, Ahmadzadeh *et al*. [21] reported that mastitis or other diseases did not prolong the DTFI. However, in this study, the cows were subjected to estrus synchronization during early lactation (21-28 days post-partum). Thus, the possible reasons for lack of consensus among different reports might be attributed to differences in breeding program followed by different farms. In line with our observations, several other researchers also reported that the DO was significantly higher in CM affected cows compared to clinically healthy cows [7,22]. More SC in these cows further substantiate the lengthened breeding period in CM cows. Although we did not study the possible confounding effects of other diseases such as dystocia, retained placenta, and ketosis on reproductive performance, Ahmadzadeh *et al*. [21] reported that mastitis alone decrease the reproductive efficiency and occurrence of other diseases had additive effects with mastitis.

### Effect of repeated or multiple episodes of CM on fertility parameters

In this study, cows diagnosed with single episode of CM had significantly (p<0.05) higher DTFDH and DTFI while DO and SC were higher (p<0.05) in multiple episodes mastitic cows compared to those diagnosed with single episode or clinically healthy cows. Several researchers [23,24] suggested that period of lactation modulates the impact of mastitis. Moussavi *et al*. [25] reported that more episodes of mastitis in early lactation significantly increased the SC with no apparent impact on the DO. Interestingly, we also observed that cows in the first and second parities diagnosed with CM had significantly delayed DTFDH. Therefore, detailed studies on effect of different type’s mastitis episodes on reproductive performance are warranted.

### Effect of severity of CM and time of occurrence of CM on reproductive parameters

We observed that a majority of cases of CM were mild to moderate in severity, which is in agreement with earlier reports [26-28]. More deviation in fertility parameters in the severely affected cows in this study could be due to the local and systemic effects of endotoxin [29]. Although effects of severe cases of CM on reproduction are less documented, greatest loss of milk production [27] and lesser pregnancy [30] in severe cases of mastitis would substantiate the observed effects. Effects of CM occurrence during different period of early postpartum was already reported by several workers [9,31,32]. DTFI was delayed when CM occurred before first AI (by 36 days than control) than the occurrence of CM after first AI (by 28 days than control) as observed by other researchers [7,20,33]. Insufficient follicular development and consequent decreased steroidogenesis, behavioral estrus; delayed ovulation or anovulation could be the possible reasons for delayed DTFI in these cows. In contrast to DTFI, DO was significantly higher in cows diagnosed with CM after first AI but before its pregnancy confirmation (by 196 days than control) than CM before first AI (by 87 days than control), which is in agreement with the previous findings [7,20,22,33]. Collectively, these findings suggest that occurrence of CM during breeding period had more adverse effects than any other periods during early lactation. Premature luteolysis and consequent loss of progesterone and thus early embryonic mortality are the suggested reasons for prolonged DO or more SC in these cows [33]. In fact, more abortion or early embryonic mortality was reported by several workers [1,8,34] when CM occurred between first AI and pregnancy confirmation.

We found that occurrence of CM beyond 62 DIM had more negative impact on reproductive outcomes in terms of DO compared to occurrence of CM during the first 4 weeks of postpartum, which is in agreement with the findings reported by Nava-Trujillo *et al*. [9]. Ahmadzadeh *et al*. [21] also reported that cows experienced mastitis before end of the voluntary waiting period had greater SC and DO, suggesting that occurrence of CM before first AI can have a carryover effect on the reproductive performance. Lavon *et al*. [11] also stated clearly that a CM event diagnosed during the first 10 days before first AI depresses fertility, and that this effect is less pronounced if the event occurs earlier than that. Hudson *et al*. [12] also found that mastitis before AI was generally associated with lesser effects on fertility. We also found that when CM occurred before first AI and it did not seriously affect the conception rate (75% of the animals conceived after second AI); however, when CM occurred during the breeding period, it had a negative impact on conception rate (data not presented). Our findings and those reported by other researchers collectively indicate that the carryover effects of CM were more significant when CM occurred during the breeding period rather than during voluntary waiting period.

### Effect of season and parity of CM occurrence on reproductive parameters

Although reports on seasonal influence of CM occurrence on subsequent fertility parameters are not available, incidence of CM during different seasons was available in India [35] or elsewhere [36]. They reported that the incidence of mastitis was highest in winter followed by summer, and thus it could be the reason for more compromised reproductive performance in this study. The adverse effects of heat stress on reproductive function is known phenomenon, and in fact, several *in vitro* [37,38] and *in vivo* [39,40] studies revealed that the elevated body temperature may directly alter the developmental competence of oocyte and embryo or indirectly affect reproductive performance through decreased feed intake and body condition [41]. When the fertility parameters of this study were analyzed between CM affected primiparous and multiparous cows, higher DTFDH was observed only in first and second parity cows. However, DTFI, DO, and SC were similarly altered in primiparous and multiparous cows. Our findings are in agreement with Nava-Trujillo *et al*. [9] who suggested that lesser energy balance, poor dry matter intake, greater loss in body condition, lesser concentration of blood glucose, insulin and insulin like growth factor-1 in primiparous could be the possible reasons for delayed DTFDH. The results had shown significant effects of CM on fertility parameters. However adjusted effects of CM on these fertility parameters after accounting for combined effects of confounding factors such as parity, stage of lactation, season and subclinical infections, could not be assessed. This was mainly due to limited number of cows and lack of subclinical infections records in these animals. Therefore, further studies, with large number of samples with suitable statistical model are necessary to understand the accurate effects of CM on reproductive performance in Indian dairy cattle.

## Conclusion

CM-affected crossbred cows had higher DTFDH, DTFI, DO and SC compared to clinically healthy cows. The negative effects of CM on reproduction parameters were higher when CM occurred during the breeding period. Further, the magnitude of adverse effects was high when CM occurred between postpartum insemination and pregnancy confirmation.

## Authors’ Contributions

NK and AM contributed equally to the study. NK and AM conducted the research work. NK, AM, MS, AK, SJ, PM and DR conceptualized the study and wrote the manuscript. LS assisted in collecting and compiling the resource material and in manuscript preparation. All authors read and approved the final manuscript.
